# The effect of various bulk filling techniques on the mechanical and structural characteristics of class I biomimetic composite dental fillings

**DOI:** 10.1038/s41598-024-81081-y

**Published:** 2025-01-05

**Authors:** Alexandra Kemény, Péter Zoltán Farkas, Borbála Leveles, Levente Borhy, Dóra Károly, Tamás Bubonyi, András Volom, Gábor Braunitzer, David S. Alleman, Balázs Varbai

**Affiliations:** 1 Department of Materials Science and Engineering, Faculty of Mechanical Engineering, Budapest University of Technology and Economics, Műegyetem rkp. 3, Budapest, H-1111 Hungary; 2 Dr. Volom Aesthetic Dentistry, Bokor u. 17–21, Budapest, H-1037 Hungary; 3 Medicontur Medical Engineering Ltd., Herceghalmi út 1, Zsámbék, H-2072 Hungary; 4 Institute of Physical Metallurgy, Metalforming and Nanotechnology, University of Miskolc, Egyetemváros C/1, Miskolc, H-3515 Hungary; 5 dicomLAB Dental Ltd, Szent-Györgyi Albert utca 2, Szeged, H-6726 Hungary; 6 Alleman Center of Biomimetic Dentistry, 3651 N 100 East Street, Suite 200, Provo, UT 84604 USA

**Keywords:** Biomimetics, Composite dental filling, Microtensile bond strength test, Class I cavity, X-ray computed tomography analysis, Fracture analysis, Biomedical materials, Biomedical materials, Imaging techniques, Scanning electron microscopy, Characterization and analytical techniques

## Abstract

Tooth degradation develops easily over time from initial caries to deeper cavities. The quality and durability of restorations are crucial in a patients’ life, and it is primarily determined by the mechanical properties of the restorations. Clinicians and engineers apply biomimetic science in dentistry to mimic the natural design of the teeth and emphasize careful material selection as well as bond strength between the tissue and artificial material. The aim of this research was to develop and compare five different bulk filling restoration techniques for high C-factor (5.0) Class I cavities and determine the most durable technique. Tensile bond strength between the dentin and the filling, as well as the inner structure of the filling was measured via computed tomography, scanning electron microscopy and mechanical testing methods. It was found that the semi-direct bulk filling technique (bulk filling with an extra, indirect curing step) can increase the tensile bond strength on average by 58% (51.1 ± 12.2 MPa) compared to the direct bulk filling method with the same materials (32.4 ± 8.2 MPa). The introduced semi-direct technique allows bulk filling to be used on Class I cavities while achieving a high bond strength, resulting in highly durable and reproducible restorations.

**Supplementary Information:**

The online version contains supplementary material available at 10.1038/s41598-024-81081-y.

## Introduction

In deep cavity restorations, dental professionals and researchers aim to create a combination of an adhesive system, filling material and technique that reach or exceed the properties of the dentin enamel junction (DEJ). Therefore, several studies have investigated the properties of the DEJ of healthy human teeth. According to Urabe et al.^1^, the tensile strength of the DEJ is 51.5 MPa. Giannini et al.^2^ measured similar results of the tensile strength of the DEJ, with the result of 46.9 ± 13.7 MPa. Later, Yamada et al.^3^ investigated the anisotropic properties of the DEJ and found that under perpendicular tensile loading, the strength of the DEJ is only 20.6 ± 4.8 MPa. Under parallel tensile loading, the value was higher, with 30.9 ± 3.3 MPa. The abovementioned results show that the measured values depend not only on the individual tooth but also on the load type and force direction to the DEJ.

Armstrong et al.^4^ published a review with the guidelines of the microtensile bond strength (µTBS) tests of dental composite bonding effectiveness, creating a useful database on the possible sample preparations and their differences. Sano et al.^5^ also wrote a review on µTBS testing a few years later, with additional measurements on the relationship between the remaining dentin thickness and the µTBS. However, the correlation was poor; the results have proven that the longer restored teeth are stored in water (24 h to 1 year), the lower the µTBS values. Similar conclusions have been found in numerous other publications^6–8^.

Furthermore, it is already known that the µTBS depends on the configuration factor (C-factor) of the restoration. The lower the C-factor, the greater the bonding force between the filling material and the tooth tissue. The C-factor refers to the number (or area) of bonded surfaces in tooth restoration and is equal to the ratio of bonded to non-bonded surfaces. As the adhered dental filling materials are usually made of some kind of resin-based polymer, which need to be applied in liquid state, and polymerized in situ, making it inevitable of shrinking due to the polymerization process. Therefore, tension forces can occur near the cavity walls during tooth restoration, which can have both short- and long-term effects and correlate with the cavity design (configuration). The cavities are also classified according to their depth and the number of sides of the cavity surrounded by a wall. Class I cavities consist of five bonded walls of the cavity and one free side; therefore, the highest C-factor restorations achieve the lowest bond strength^9,10^. As a solution, some research is based on the development of tooth restorations by restoring Class I deep fillings with a low C-factor. This is called micro C-factor treatment and involves applying the composite in thin layers of ~ 1 mm and polymerizing it so that the largest surface area dominates the bond strength. Unfortunately, this is a very lengthy method for everyday application compared to the oblique layering used by dentists, so other stress reduction techniques are needed.

Vasconcelos Monteiro et al.^11^ concluded that the bulk filling of Class I cavities allows for faster restorative treatments, reducing the time required for cavity filling and can also improve the immediate surface and marginal quality of the fillings. Leinonen et al.^12^ recently measured the time-saving of the bulk fill base technique to fill a cavity compared to the conventional incremental technique, resulting in time savings of approximately 59.8% or 4 min and 36 s. Medina-Sotomayor et al.^13^ found similar results with a restoration time of 3.52 min for the bulk fill technique, and a significantly smaller internal gap for bulk fill composite resins (63.31 μm) compared to conventional resins (333.14 μm).

However, bulk filled materials may exhibit significant polymerization shrinkage, which affects the overall physical properties of the material^14^. Gjorgievska et al.^15^ used microcomputed tomography (µCT) and scanning electron microscopy (SEM) to investigate the potential gaps between dental fillings and the cavities and found that the low-viscosity flowable bulk fill composites exhibited better adaptation, higher efficiency of polymerization, and lower porosity compared to sculptable bulk fill composites.

The dentin-filling bond can be characterized by its µTBS^16,17^, shear strength^18^ or bending properties^19^. There are some µTBS results of Class I bulk filled restorations with various materials. Nikaido et al. evaluated the bulk restorations of Class I (C = 4.0) cavities with the use of LB 2 V adhesive and AP-X composite. They used cyclic thermal and mechanical loads before the µTBS tests and found that the bond strength decreases up to 23% on average with the increasing cyclic loads. Nikolaenko et al.^10^ restored Class I (C = 5.0) cavities with three different dentin adhesives and 10 different filling techniques. The bulk filled restorations showed significantly lower µTBS (with a maximum of 11.8 ± 8.8 MPa) than the layered ones. Ende et al.^20^ prepared Class I cavities with two different C-factors (C = 5.6 and C = 3.9) for bulk filling restorations. G-ænial Bond was applied to the dentin for all specimens, and the highest µTBS (33.9 ± 11.8 MPa) was reached with the filling intended for a maximum of 4 mm layer thickness by the manufacturer (SureFil SDR Flow); therefore, this product was suitable for bulk filling. In another research, Ende et al.^21^ prepared Class I cavities (C = 5.8) and investigated two additional bulk fill composites (Filtek Bulk Fill, Tetric EvoCream Bulk Fill) to the previously applied SureFil SDR Flow. The µTBS results showed a decrease in the values compared to their previous study, but the highest values were reached using the SureFil SDR Flow again (16.6 ± 7.7 MPa). Han and Park^22^ investigated the µTBS of Class I (C = 5.0) cavities with two different bulk fill composites (Tetric N-Ceram Bulk Fill, Venus Bulk Fill), bonded by Clearfil SE Bond and restored by the bulk filling technique or the conventional incremental technique. The results showed a significant decrease (more than 50%) in the µTBS of the bulk filled restorations compared to the conventional method.

Sadr et al.^23^ created deep Class I restorations (C = 6.3) in resin composite moulds for optical coherence tomography (OCT) gap analysis and in human teeth for µTBS. Clearfil SE Bond 2 adhesive system was used and SDR bulk fill composite with different techniques. The bulk filled restoration showed the largest gap volume at the cavity floor, which was eliminated by using UHMWPE Ribbond fiber. The incremental technique resulted in the highest µTBS at 31.7 ± 12.5 MPa.

In the above-mentioned papers, the attainable µTBS of the high C-factor restorations was lower with the bulk filling method compared to the conventional incremental technique. Therefore, the bulk filling method is fast, but there are disadvantages due to the polymerization shrinkage, causing reduced bond strengths. Therefore, the bulk fill materials are the solutions of the manufacturers for managing high C-factor cavities; however, they only consider the dentist’s time and not the patient’s interests. The patient will not be aware of the compromises involved in receiving such a filling – specifically, that the material will inevitably shrink somewhat during polymerization. This generates stress that can create cracks or gaps along the filling, which reduces the bond strength, potentially resulting in secondary caries, which is not beneficial for long-term healthy teeth.

The aim of this research was to create a high tensile bond strength bulk filling-based restoration and, therefore, to find a solution for stress reduction while maintaining the possibility of fast restoration time. In the experimental section, the properties of Class I biomimetic composite dental fillings have been extensively characterized, and the effect of various bulk filling techniques on the bond strength, fracture surface and gas inclusions inside the fillings were determined.

## Experimental section

**Fig. 1:**
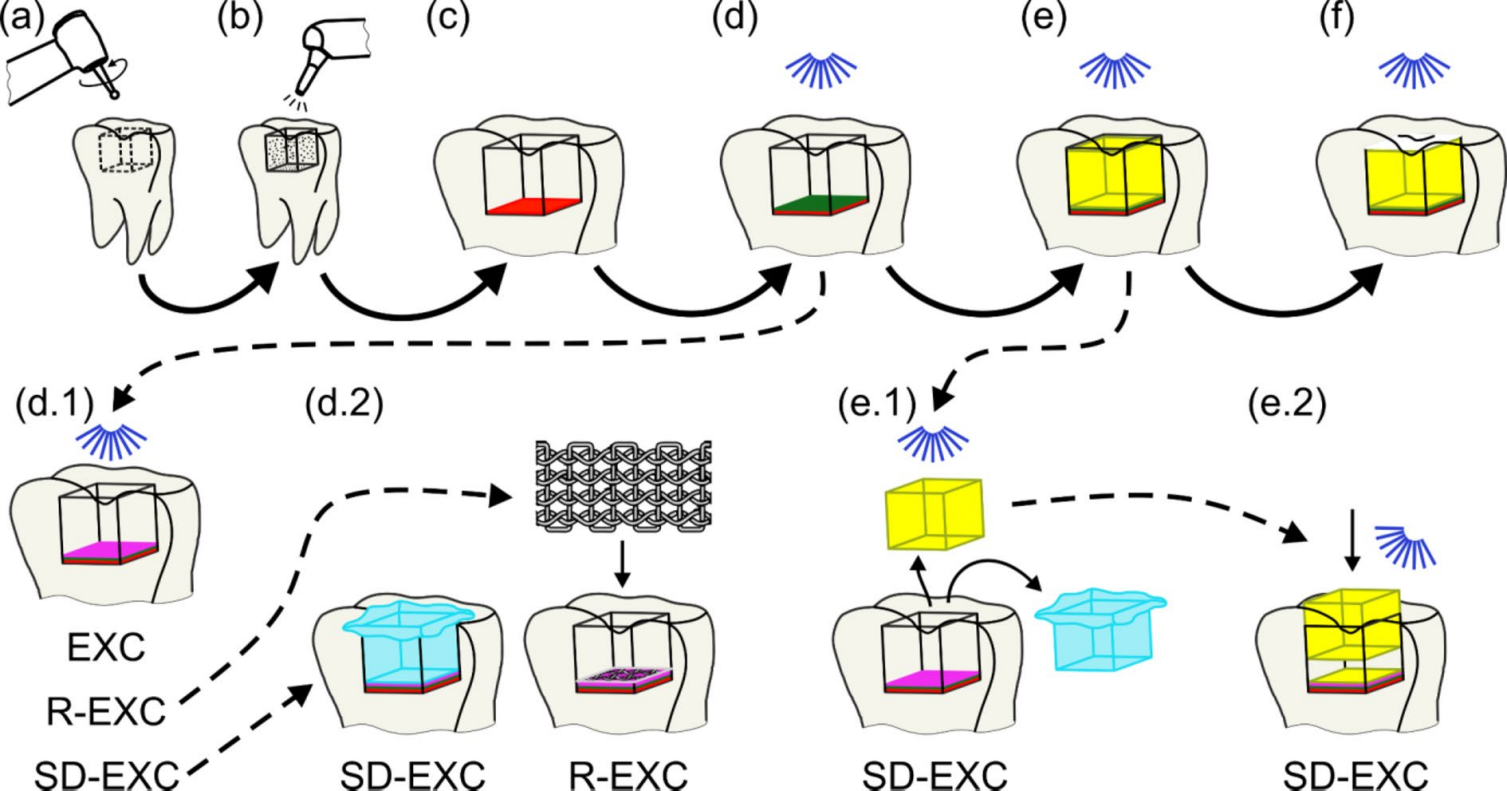
The restoration steps for each type of specimen. Preparation of the Class I cavity (**a**), air abrasion (**b**), application of the primer (**c**), application of the adhesive (**d**), bulk filling (**e**) and enamel restoration (**f**). Additionally, for specific sample types: additional composite layer on the adhesive (**d.1**), application of Teflon foil or Ribbond strip (**d.2**), indirect polymerization (**e.1**), and the reinsertion of the filling (**e.2**).

A total of 10 healthy third molars were used in this research, extracted due to orthodontic treatment reasons from persons aged 22 –55. All methods were performed in accordance with the relevant guidelines and regulations as stated in the *Ethical approval* section. A Class I, 4 × 4 × 4 mm cavity was prepared in each specimen (Fig. 1a), aiming for the highest residual stresses. The cavities had a C-factor of 5.0, calculated from the ratio of the adhered surfaces and the free surface.

### Applied dental filling techniques

The surfaces of the Class I cavities were treated with 27 μm particle size alumina air abrasion for 15 s with 500 kPa pressure from 2 to 4 mm distance (Fig. 1b), which helps the adhesive to build a higher strength due to the increased surface area, while also creating compressive stress in the dentin, resulting in a higher resistance against crack formation and propagation. It was also previously found that air abrasion helps to compact and thin out the smear layer formed during preparation, further increasing the bonding strength in the case of self-etch bonding systems^24^. The air abrasion was executed with a Rondoflex handpiece (KaVo Dental Co., Biberach, Germany). Then, the rim of the enamel was etched with 37% orthophosphoric acid for 30 s. As the primer and adhesive at the bottom of the cavity, Clearfil SE protect (Kuraray Co., Ltd., Tokyo, Japan) was used, which is a light-cured, self-etch two-step adhesive system. The primer was applied for 30 s by rubbing the bottom of the cavity, with an additional 20 s to evaporate the solvent (Fig. 1c). The adhesive was applied in one thick layer, and the excess was removed by suction prior to 20 s of light curing at 476 nm and 1400 mW/cm^2^ (Fig. 1d). A VRN VAFU LED lamp (Guilin Veirun Medical Technology Co., Ltd., Guangxi, China) was used for all polymerizing steps.

It has been previously found that an oxygen inhibition layer is forming on top of the adhesive, which prevents the polymerization of adhesive resins up to a thickness of 10–20 μm^25,26^. To counterbalance this effect, an additional thin layer of Clearfil Majestic Flow (Kuraray Co., Ltd., Tokyo, Japan) composite was applied on the adhesive in a 0.5 mm thickness and cured for 20 s (Fig. 1d.1). The positive effect of this additional layer was deducted from the comparison of two almost identical sample types differing only in the Clearfil Majestic Flow composite layer.

Bulk filling of Class I cavities creates the highest polymerization shrinkage stresses. However, the shrinkage can be minimalized using biomimetic composite filling materials^27^. All cavities have been restored with the bulk filling method in one step and cured for 20 s (Fig. 1e). EverX Flow (GC Corporation, Tokyo, Japan) has been used as a dentin-replacement glass fiber-reinforced polymer. The enamel has been restored by covering the dentin-replacement with the Asteria Estelite OCE (Tokuyama Dental Corp., Tokyo, Japan) composite in 4 layers, cusp by cusp, cured for 20 –20 s from all sides (Fig. 1f). The specimens were stored at room temperature in 0.5 wt% Chloramine-T solution to avoid dehydration.

Firstly, as a universal reference (‘ref’), G-ænial A’CHORD (GC Corporation, Tokyo, Japan) composite has been used with a bulk-filling method, without the Clearfil Majestic Flow layer. The second sample type was bulk filled with the biomimetic EverX Flow, without the Clearfil Majestic Flow layer, named as ‘EX’, while the third sample type consisted of the same technique and EverX Flow filler but with the additional (Clearfil Majestic Flow) composite layer to counterbalance the oxygen inhibition effect, named as ‘EXC’. In the fourth sample group, a semi-direct bulk filling technique was applied (named as ‘SD-EXC’). The semi-direct filling technique is a method that combines elements of both direct and indirect restorative techniques. In this approach, part of the restoration is performed extraorally (as in indirect techniques), but the final placement and bonding happens directly in the oral cavity. For this purpose, the EverX Flow was placed into a cavity covered in Teflon foil (Fig. 1d.2), where the composite has been cured for 20 s. After that, the filling was removed from the cavity and polymerized again outside from every direction (Fig. 1e.1). The Teflon foil was removed, and the filling block was reinserted into the cavity with additional EverX Flow for adhesion and gap closure, where the filling was again cured for 20 s (Fig. 1e.2). The semi-direct technique should further decrease the residual inner stresses of the filling, resulting in a potentially higher strength bond with the dentin. However, it is essential to note that there are geometric limitations of the technique, as it is not applicable if an undercut is formed within the tooth during the drilling preparation. The fifth and final sample type was the technique where a 3 mm wide Ribbond (Ribbond Inc., Seattle, WA, USA) UHMWPE strip was placed on top of the cured Clearfil Majesty Flow layer into a thin layer of AP-X composite (Kuraray Co., Ltd., Tokyo, Japan). The layer was then drenched in adhesive and cured for 30 s before bulk filling the cavity by the EverX Flow composite (Fig. 1d.2). Table 1 summarizes the prepared and investigated sample types, highlighting the main differences between them. Additionally, a supplementary figure (SF1) shows each used preparation technique in a step-by-step manner for the increased clarity of the process.

**Table 1:** The investigated sample types (‘X’ indicates that the listed material or technique was applied).

	Ref	EX	EXC	SD-EXC	*R*-EXC
Clearfil majesty layer	–	–	X	X	X
Ribbond	–	–	–	–	X
Direct bulk filling	X	X	X	X*	X
Indirect polymerizing	–	–	–	X	–

*The cavity was separated from the filling material with a Teflon foil.

### Structural and mechanical measurements of the restorations

**Fig. 2:**
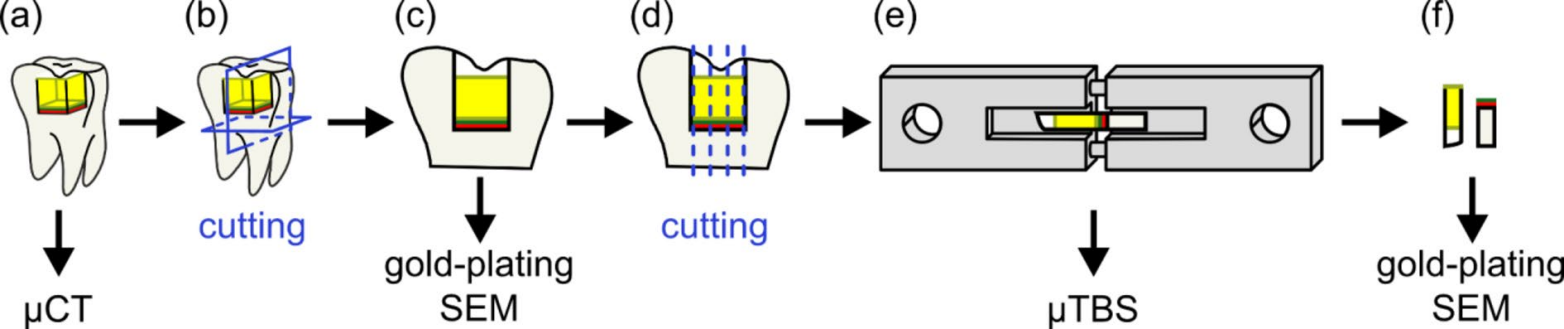
The schematic process chart shows the specimen preparation steps and measurement methods.

Micro-CT analysis was executed on the restored teeth as a non-destructive method to characterize the restorations based on the inner pore amount (Fig. 2a) on a YXLON FF35 dual-tube CT equipment (YXLON International GmbH, Hamburg, Germany). The teeth samples were placed into a high-power target X-ray for the measurements with 160 kV voltage and a 20 µA current. The detector distance was 1150 mm, and the object was 40 mm from the X-ray tube. A Varian 2530HE detector with a 1.0 Hz framerate was used for imaging. The applied parameters resulted in a voxel resolution of 9.2 μm. The samples were reconstructed and evaluated using VGSTUDIO MAX 3.3 software. The boundary between the filler and the dentin could not be evaluated precisely, as the thin adhesive layer and the X-ray imaging noise made it impossible to measure; therefore, additional measurement methods were needed to evaluate the adhesive layer.

For further measurements, the specimens were prepared by cutting with a Buehler IsoMet 1000 diamond disc precision cutter (Buehler Ltd., Lake Bluff, IL, USA). First, slices were cut to 1 mm thickness perpendicular to the occlusal plane (Fig. 2b) for scanning electron microscopy (SEM) imaging. This was aimed to support the CT analysis results and give additional information on the possible gaps between the dentin and the restoration. The SEM imaging was done with a Zeiss EVO MA10 (Carl Zeiss AG, Jena, Germany) microscope with 20 kV accelerating voltage, with a secondary electron (SE) detector after gold-plating the measured surface of the slices (Fig. 2c). After the measurements, the slices were cut into a total of 9 pieces of 1 × 1 mm base blocks (Fig. 2d) for µTBS tests per tooth to numerically evaluate the bond strength of the restorations under tensile loads. Detailed information on the specimen preparation method has been provided in a previous article by the authors^28^.

The µTBS tests were carried out on an Instron 5965 (Instron, Norwood, MA, USA) electromechanical universal material testing machine under quasi-static conditions with 1 mm/min crosshead speed, with the aid of stainless-steel jigs aligned with pins that restrict bending and torsional loads (Fig. 2e). The block specimens were attached to the jigs with a 3 M Scotch-Weld PR100 Instant Cyanoacrylate Adhesive (3 M, St. Paul, MN USA), based on the findings in previous research of the authors^29^. The tensile bond strength was calculated as the ratio of the measured maximum force and the original cross-section area.

The fractured specimens were gold-plated and investigated by SEM (Fig. 2f) to determine the exact location of the fracture in the structure of the dentin-filling system.

## Results

### Porosity and structure analysis

**Fig. 3:**
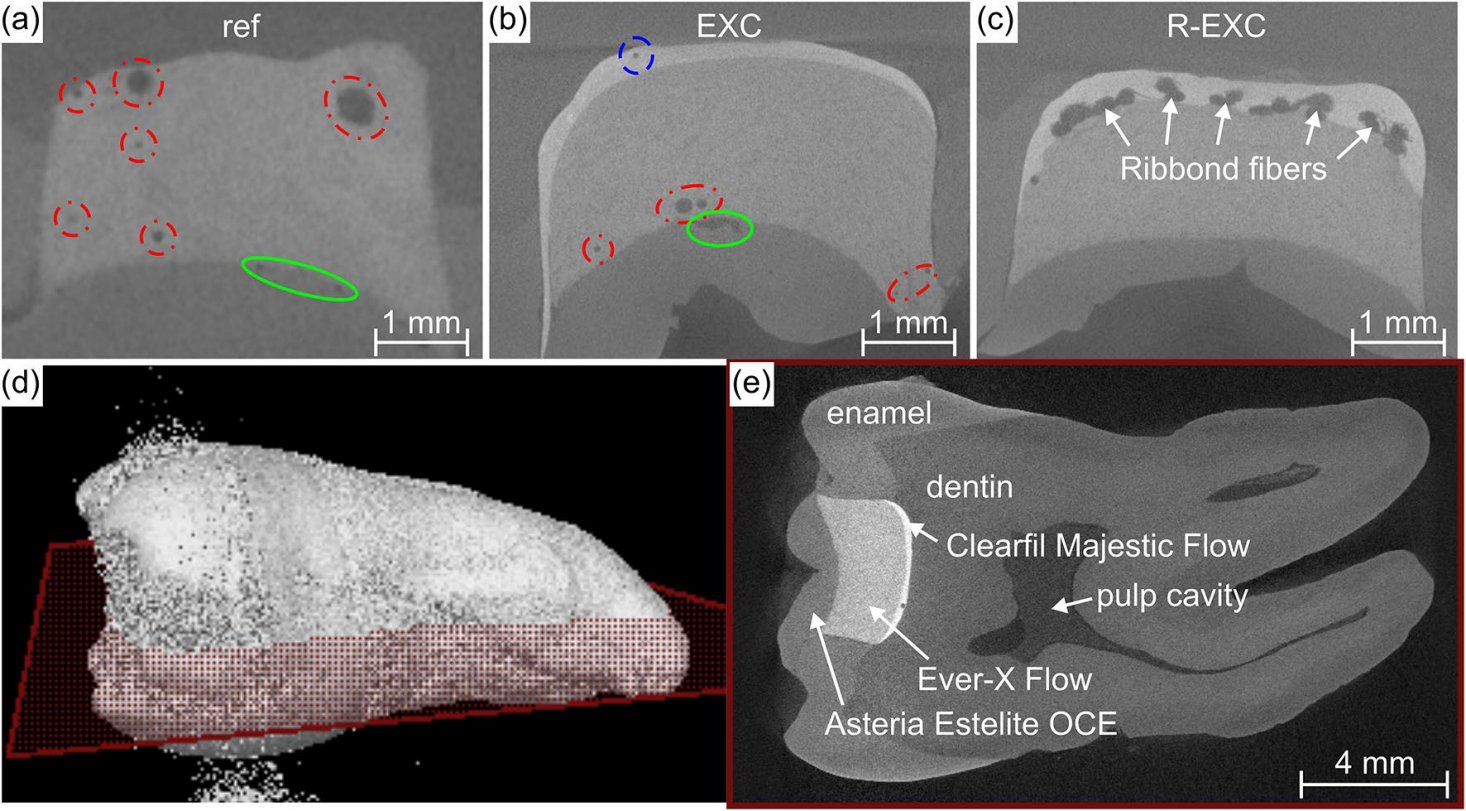
µCT slice of inclusions at various locations within the restoration (**a**) and (**b**), the Ribbond fibers within the AP-X composite layer (**c**), a 3D reconstructed tooth (**d**) and the marked slice of the tooth, with the assessable layers labelled (**e**). The dashed blue circle shows an inclusion in the Clearfil Majestic Flow layer (**b**), the red dash-dotted circles mark inclusions within the bulk-filled G-ænial A’CHORD (**a**) and Ever-X Flow (**b**) composite near the enamel replacement layers, and finally, the green solid circles represent inclusions in the Asteria Estelite OCE layer (**a**) and (**b**).

From the CT measurements, the total volume of inclusions was determined numerically, along with their location characterized (Fig. 3a and b). The average inclusion size of the Ever-X bulk filled samples were of 8.06·10^− 4^ mm^3^, while the same value for the G-ænial A’CHORD bulk filled samples was 1.53·10^− 4^ mm^3^. However, the high number of relatively small inclusions for the reference type added up to a total inclusion volume, that was on average 57% higher than the other types. With X-ray imaging, resulting in a black and white picture, the Ribbond fibers are shown with a similar grayness level as the gas inclusions (Fig. 3c). Thus, the R-EXC samples were excluded from the numerical characterization, as the Ribbond fibers could not be separated visually from the inclusions. Figure 3d and e show the 3D reconstruction of an investigated tooth and a slice with the various layers shown, respectively.

**Fig. 4:**
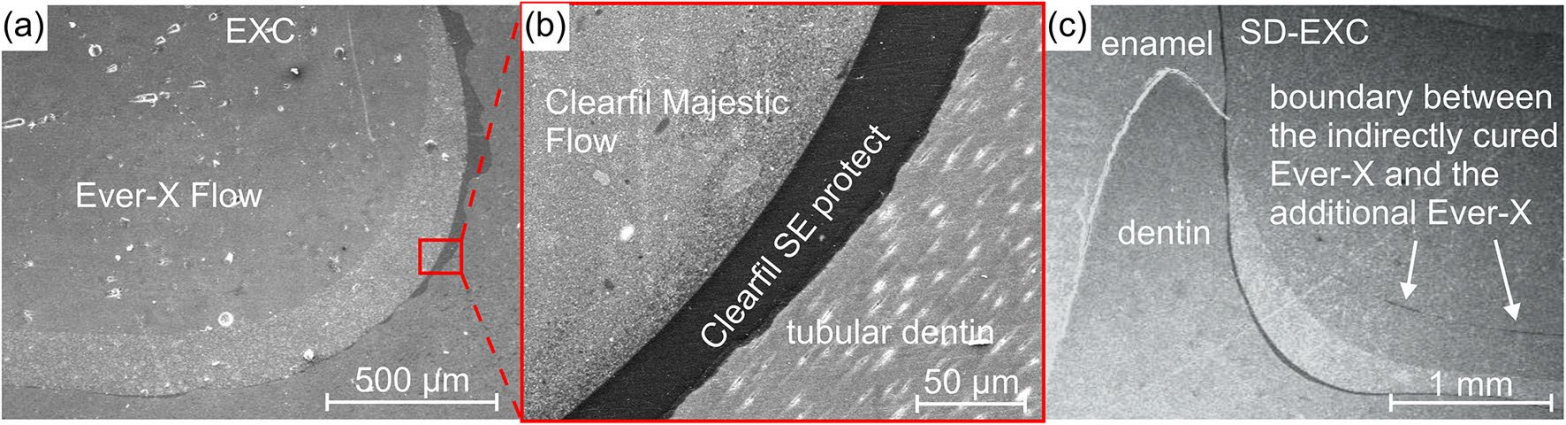
SEM images of the restoration structure. Image of an EXC slice in lower (**a**) and higher (**b**) magnifications and the visible boundary in the SD-EXC slice between the indirectly cured Ever-X Flow and the additional Ever-X Flow (**c**).

SEM images support the further analyzation of the sample structures with higher magnifications and better resolution. The layers of the tissue and fillings can be identified on Fig. 4. Higher resolution pictures of the EXC and the SD-EXC samples gave additional information to the CT results about their boundary region in high magnification; which are included in Fig. 4. The adhesive layer thickness was not homogeneous, as seen in Fig. 4a and c, due to the manual suction removal of the excess adhesive.

### Microtensile bond strength

**Fig. 5:**
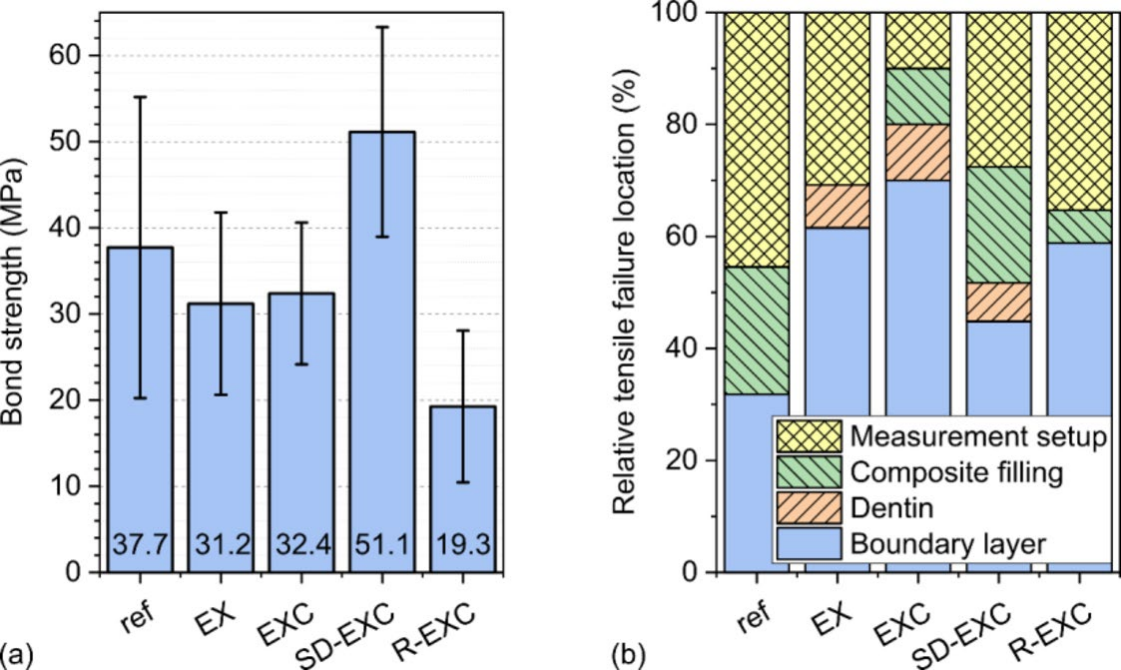
The results of the µTBS tests (**a**) and the relative tensile fracture location amounts of each sample type (**b**).

The results of the µTBS tests are shown in Fig. 5a. After visual investigation, only the values of those pieces were included where the failure occurred at the boundary layer (Fig. 5b). Dominantly, the semi-direct technique (SD-EXC) resulted in the highest tensile bond strength values, while the second highest average tensile bond strength surprisingly belonged to the reference sample type with a large deviation.

The two differently prepared direct bulk-filled EverX groups (EX and ECX) showed similar results. Finally, the lowest tensile bond strength belonged to the application of the Ribbond strip (R-ECX).

### Fracture properties

Regarding fracture, only 50% of the samples split at least partially at the boundary layer between the dentin and the filling. The rest were excluded from tensile bond strength evaluation as the fracture occurred either within the dentin tissue, the filling material or the specimen was detached from the jig. Figure 5b shows the relative failure locations for all sample types.

**Fig. 6:**
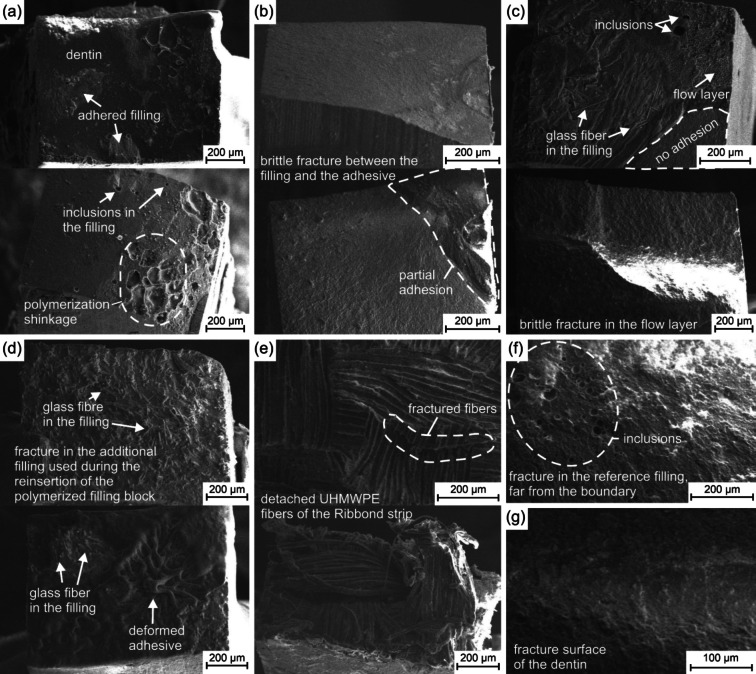
The fracture surfaces of the different sample types by SEM. Ref (**a**), EX (**b**), EXC (**c**), SC-EXC (**d**), R-EXC (**e**), fracture in the ref filling (**f**) and fracture in the dentin (**g**).

The fracture properties were further investigated via SEM, presented in Fig. 6. Under the microscope, the different fracture locations and mechanisms could be easily distinguished.

## Discussion

The fracture mechanisms of differently bulk-filled teeth are assessed. Each fracture mechanism can be described by its value (tensile bond strength), location (where the filling system split), and method (the trajectory of the fracture). Each failure gives us information about the bond strength and, thus, the longevity of the restoration itself.

Due to the uniqueness and the manual preparation of the teeth, each fracture showed an individual fracture mechanism. This is the reason behind the large deviations in µTBS results and the different appearance of the fracture surfaces. However, strong findings of this research suggest that a novel protocol (named Semi-Direct) surpasses all the other compared techniques for bulk filling.

The location of the failure is first determined visually and via SEM. The executed tensile testing results show that about 50% of the samples split at the desired adhesive boundary between the dentin and filling, which was the target of our measurements. During tensile loads, inclusions, material differences, small cracks, and other imperfections will act as stress concentration points and might be the starting point for failures and, finally, detachment. If the failure has occurred far from the boundary, two main mechanisms could be distinguished. The fracture of the filling materials always started from either the inclusions in the block (Fig. 6f) or the glass fibers perpendicular to the loads, as they act as stress concentration points (similarly to Fig. 6c top or d). If the fracture occurred in the dentin, it was always brittle, starting from a stress concentration point (e.g. the tip of a crack). See Fig. 6b. The relatively low adhesive failure implies that there might be other weak points in the system (e.g. measurement setup, dentin imperfections or glass fibers, inclusions in the composite). The number and size of inclusions measured with CT should give us valuable predictions of possible failure mechanisms. This finding relates to the tensile testing results for the reference sample. When a large number of inclusions were measured, the tensile bond strength was low, while with no inclusions, the tensile bond strength was high. This is somewhat incoherent for dependable bulk filling. Naturally, other environmental factors also might influence the filling, and vacuum naturally cannot be used before curing inside the mouth to eliminate the gas voids.

SEM and CT images of samples slices are very similar to each other; however, SEM could achieve a significantly higher magnification if needed. For the purpose of this research only the SEM images of the EXC and the SD-EXC samples presented additional information to the CT results, shown in Fig. 4. Figure 4b of the EXC shows the magnification of the boundary between the dentin and the restoration, with the visible adhesive layer, and that there are no micro-gaps present. The adhesive layer thickness was not homogeneous, as seen in Fig. 4a and c, due to the manual suction removal of the excess adhesive. This could have a positive effect with enlarging the adhesive surface area, but it could also lead to uncontrolled adhesion. The thickness of the adhesive layer was measured by the authors in a previous publication^28^. The SD-EXC sample (Fig. 4c) showed the boundary region with a barely visible boundary layer between the additional layer of Ever-X Flow and the extraorally cured and reinserted Ever-X Flow block. This might be a considerable stress concentration point for tensile loads.

Due to manufacturer recommendation, it was expected that the Ever-X Flow composite (intended for bulk filling) will yield significant tensile bond strength results. However, our findings suggest that there is still significant polymerization shrinkage present, which an indirect curing step might eliminate. Using the semi-direct technique, the measured bond strength achieved a dominantly higher value compared to the other methods. This is a significant result, presenting a novel preparational protocol, for which only one extra tool is needed: the Teflon foil. As presented in the results, the achieved tensile bond strength was the highest (51.1 ± 12.2 MPa) for the Semi-Direct (SD-EXC) restorations, which is comparable with the highest measured tensile strength of the DEJ of healthy human teeth (51.5 MPa, according to Urabe et al.^1^). Considering that the SD-EXC has reached the highest TBS, their fracture should also differ from the other types. The fracture always occurred at least partially in the EverX filling with the detached glass fibers visible on the SEM images (Fig. 6d); however, it cannot be determined visually whether the fracture happened by the boundary or it started from a random inclusion or glass fiber in the Ever-X Flow material. In some cases, the adhesive boundary layer (seen in Fig. 4c) suffered plastic deformation (Fig. 6d bottom).

The second highest average tensile bond strength surprisingly belonged to the reference sample type with a large deviation of 37.7 ± 17.5 MPa. These restorations either resulted in high or very low strength values, depending on the quality of the samples, as the filling material is not intended for bulk filling; therefore, it either contains a high number of pores or none at all. Analyzing the SEM images of the fracture surfaces show, that the reference samples had significant polymerizing shrinkage, and additional inclusions were found in the filling (Fig. 6a bottom). However, the relatively high TBS values are supported by the findings that the filling adhered well to the dentin, even without an additional composite layer to counterbalance oxygen inhibition (Fig. 6a top). This material might have high adhesive properties, but as mentioned previously it is not consistently dependable for bulk filling; however, it might be a good choice for traditional incremental filling for Class I cavities.

In the Ever-X Flow bulk filled samples the obstruction of the formation of the oxygen inhibition layer resulted in a slight increase in the tensile bond strength from 31.2 ± 10.6 MPa (EX) to 32.4 ± 8.2 MPa (EXC). The results show that the extra layer does not have a significant influence on pure tensile loads. Nevertheless, it may have a more notable outcome on other types of loads, such as shear, (cyclic) compression, or bending. Even though the EX and EXC samples only had a small difference between their TBS results, their fracture mode varied significantly. Within the EX specimens, the fracture usually occurred between the adhesive and the filling with a brittle surface, showing that the oxygen inhibition layer prevented bonding to the EverX (Fig. 6b). On the other hand, if an additional flow layer was used (EXC), a stronger bond was formed between the EverX filler and the dentin, as the fracture mainly occurred within the EverX filling material (see the fibers in Fig. 6c top) and the Majesty Flow material. This phenomenon can be the result of the successful counterbalance of oxygen inhibition. The results align with the findings of Dall’oca et al.^30^, as the oxygen inhibition did not influence the TBS results significantly but changed the fracture properties of the specimens. The fracture was only partially brittle, where no adhesion was formed (Fig. 6c). However, the fracture rarely occurred within the Majesty Flow material.

As expected, the lowest tensile bond strength belonged to the application of the Ribbond strip (R-ECX) with 19.3 ± 8.8 MPa. The use of a stress-relieving high plasticity material with properties different from those of brittle dentin or filler materials will lower the pure tensile properties of the filling system. However, this material will also increase the resistance against shear and cyclic compressive loads and reduce the inner stresses induced by the polymerization shrinkage^31,32^. The R-EXCs resulted in the lowest TBS values, with a unique fracture. As the weakest part of the restoration was the UHMWPE strip for tensile loads, the failure occurred inside the Ribbond. The loads were perpendicular to the strips, so the fibers could not take the induced stress, resulting in the detachment and fracture of the woven fibers (Fig. 6e). In the future, cyclic testing of the examined protocols might present valuable data additionally to the µTBS, as it represents the true mechanical loads on the teeth better.

This research presents clinically significant findings relating bulk filling of Class I cavities, with a novel, Semi-Direct protocol, which utilizes the polymerization shrinkage stress relieving benefits of the indirect curing step. With this method the tensile bond strength of the natural boundary between two teeth tissues of the human dentin − enamel junction can be reached.

## Conclusions

The following conclusions were drawn from the research presented above on the tensile behavior of various filling techniques of Class I biomimetic composite dental fillings. It has to be noted that the used tooth sample size can affect the deviation of the results, and the uniqueness of each tooth influences the achievable bond quality.

- The highest tensile bond strength (51.1 ± 12.2 MPa) could be achieved with the semi-direct technique, reaching the tensile strength of the dentin − enamel junction, as the polymerization shrinkage of the bulk filling has been reduced with the indirect polymerizing and reinsertion of the filling block. The primary failure mode of these specimens was within the EverX composite.
- There was no significant difference between the tensile bond strength of the EverX bulk filled specimens, whether an additional layer of filling had been used to reduce the oxygen inhibition of the adhesive or not. However, the failure of the two types differed, showing that the additional filling layer helped the adhesive bond to the EverX.
- The use of the UHMWPE Ribbond strips reduced the tensile bond strength, showing that the fibers of the Ribbond, could not act as tensile load-bearing elements of dental restorations. Cyclic testing might be recommended for better evaluation.
- The reference restorations had quite high tensile bond strength, even though they had a significant number of inclusions within the bulk-filled block, as the polymerization shrinkage is larger. The high strength corresponds to the good adhesion of the filler material; however, the inclusions can act as a starting point for secondary caries, which should be prevented.

In summary, the Semi-Direct technique showed the best results in every measured aspect of the fracture mechanism, with only slightly more complexity to the direct bulk filling of the cavity. This novel protocol is applicable in every case where there is no undercut prepared during drilling. The clinical significance for dental practices in utilizing a simpler and faster method than the traditional layer-by-layer filling of deep, Class I cavities.

## Electronic supplementary material

Below is the link to the electronic supplementary material.

Supplementary Material 1

Supplementary Material 2

## Data Availability

The datasets analyzed during the current study available from the corresponding author on reasonable request.
