# Supplementary material for: The effect of various bulk filling techniques on the mechanical and structural characteristics of class I biomimetic composite dental fillings
Source: Sci Rep. 2025 Jan 5;15:862. doi: 10.1038/s41598-024-81081-y (PMC11701090; doi:10.1038/s41598-024-81081-y)
Supplement: Supplementary file 2 — Supplementary Material 2 [file 41598_2024_81081_MOESM2_ESM.docx]

Supplementary material


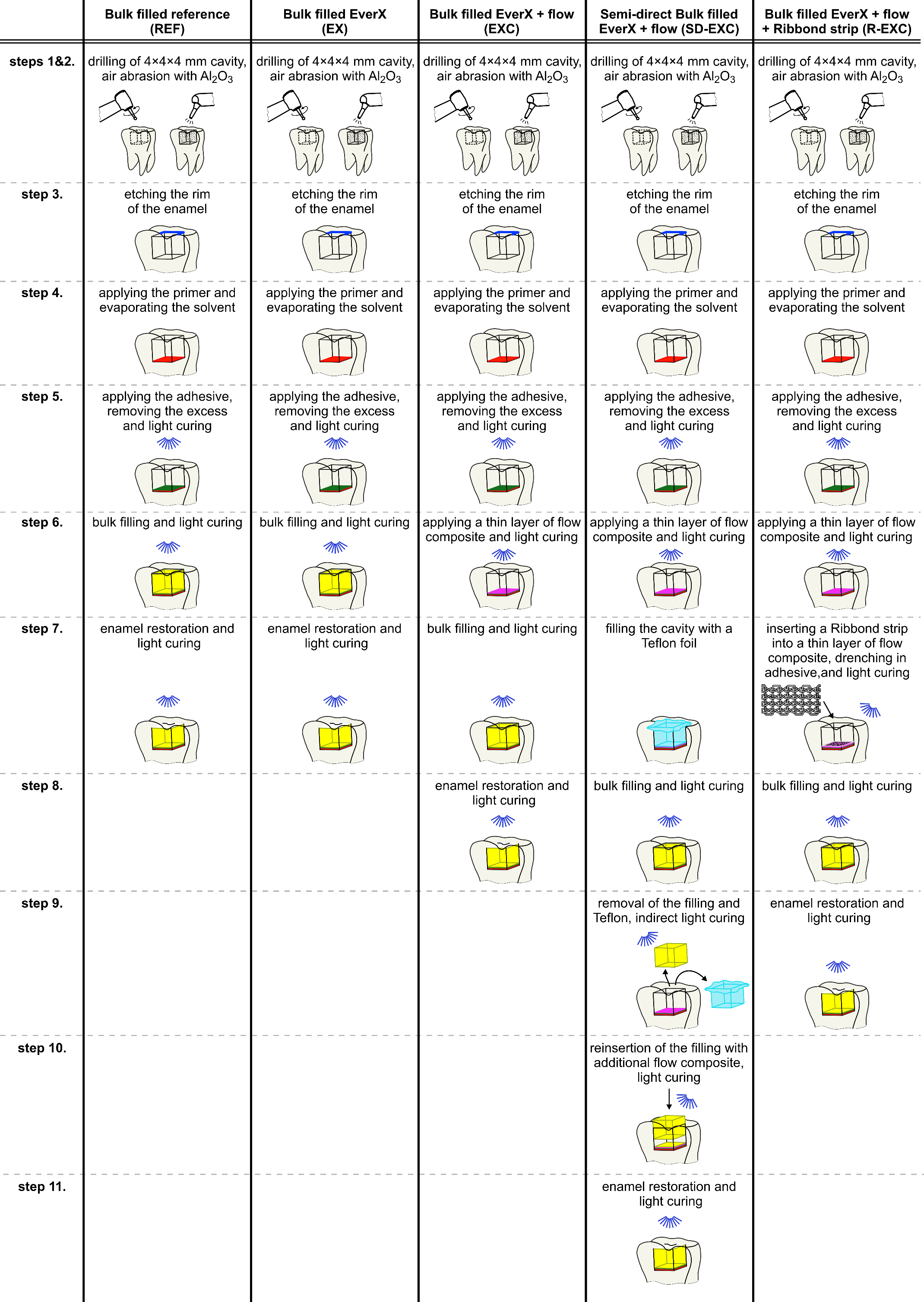


SF1. Step-by-step preparation procedure of each applied dental filling method.
